# Spontaneous (Hashimoto-like) chronic lymphocytic thyroiditis in a rhesus macaque (*Macaca mulatta*)

**DOI:** 10.5194/pb-8-37-2021

**Published:** 2021-06-01

**Authors:** Roland Plesker, Gudrun Hintereder

**Affiliations:** 1 Central Animal Unit, Paul-Ehrlich-Institut, Langen, Germany; 2 Zentrallabor, Universitätsklinikum Frankfurt, Frankfurt, Germany

## Abstract

A case of a female, 10-year-old rhesus macaque (*Macaca mulatta*) with spontaneous chronic lymphocytic thyroiditis is presented. At necropsy, the thyroid gland was slightly enlarged, with up to 2 mm large, round, confluent, beige foci on the surface of both lobes. Histopathologic features resembled human Hashimoto's thyroiditis: multifocally, the interstitium was infiltrated by lymphocytes and variably sized lymphoid follicles. In the pituitary gland, there were increased numbers of large, basophilic cells throughout the adenohypophysis. Using a human electrochemiluminescence immunoassay (ECLIA), no autoantibodies against thyroglobulin, thyroid peroxidase, or thyroid-stimulating hormone receptor were detected.

## 1 Introduction and literature

In humans, the term “thyroiditis” encompasses many relatively common thyroid disorders such as Hashimoto's thyroiditis, painless postpartum thyroiditis, painless sporadic thyroiditis, painful subacute thyroiditis, suppurative thyroiditis, drug-induced thyroiditis, and Riedel's thyroiditis. Among these, Hashimoto's thyroiditis, painless sporadic thyroiditis, and painless postpartum thyroiditis all have an autoimmune basis (Pearce et al., 2003). The mechanism for autoimmune destruction of the thyroid involves both cellular immunity and humoral immunity. Lymphocytic infiltration of the thyroid gland by equal numbers of B cells and cytotoxic T cells is a common histologic feature of all forms of autoimmune thyroiditis (Pearce et al., 2003).

Chronic lymphocytic thyroiditis (or Hashimoto's thyroiditis) is the most common inflammatory condition of the thyroid gland in humans (Guzman and Radi, 2007) and the most frequent autoimmune thyroid disorder (Ragusa et al., 2019). It is a condition characterized by high titers of circulating antibodies to thyroid peroxidase and thyroglobulin (Slatosky et al., 2000). Classically, in Hashimoto's thyroiditis, the thyroid gland is enlarged and firm. Microscopically, the interstitium of the thyroid is infiltrated by lymphocytes with some plasma cells and macrophages, as well as variable degrees of fibrosis. Lymphocytes organize into true lymphoid follicles, with topological compartmentalization of T cells in the cortex and B cells in the center, often displaying well-defined germinal centers. In some areas, thyroid follicular cells (so called Hürthle cells) are enlarged and polygonal with subtle granular eosinophilic cytoplasm and a vesicular nucleus with one prominent nucleolus (Caturegli et al., 2014).

Spontaneous and experimentally induced chronic thyroiditis has been described in a number of animal species including dogs (Haines and Penhale, 1985; Benjamin et al., 1996), rabbits (Rose and Witebsky, 1956), guinea pigs (Karesen and Godal, 1969), rats (Noble et al., 1976; Kitchen et al., 1979), multimammate mice (Solleveld et al., 1985), mice (Braley-Mullen et al., 1999), chickens (Cole et al., 1968), and turkeys (Plesch et al., 2014).

In contrast, little has been published about spontaneous thyroiditis in nonhuman primates, despite a number of publications describing experimental thyroiditis in macaques (Kite et al., 1965; Rose et al., 1965; Doebbler and Rose, 1966; Dumonde, 1966; Kite et al., 1966; Rose et al., 1966; Andrada et al., 1968; Themann et al., 1968; Andrada et al., 1973) and vervet monkeys (Pudifin et al., 1977). In a cynomolgus macaque *(Macaca fascicularis),* a case of spontaneous chronic lymphocytic thyroiditis has been described (Guzman and Radi, 2007). In this publication, a hypertrophic and hyperplastic adenohypophysis is additionally reported containing hypertrophied cells with abundant pale basophilic, sometimes vacuolated cytoplasm. These cells were strongly positive for TSH in immunohistochemistry. In addition, a case of a Hashimoto-like chronic lymphoplasmocytic thyroiditis in an African green monkey (*Chlorocebus aethiops*) was presented in the Armed Forces Institute of Pathology (AFIP) Wednesday Slide Conference in 2008 (Conference 2, 10 September 2008, Case IV-08012). It gives one of the more detailed descriptions of a chronic lymphocytic thyroiditis in nonhuman primates.

In a survey on 494 marmosets (*Callithrix* sp. and *Saguinus* sp.), Levy et al. (1972) observed 40 cases of chronic thyroiditis (8.1 %). They assumed a genetic component for the occurrence and noticed females to be more often affected. In one case of a Silvery marmoset (*Callithrix argentata*), the pathohistological changes in the thyroid mimicked Hashimoto's disease. Besides these descriptions, hints on the possible incidence of chronic thyroiditis in nonhuman primates can be found in a few necropsy surveys (Chamanza et al., 2010; Guardado-Mendoza et al., 2009; Tucker, 1984; Chalmers et al., 1983).

To our knowledge, no reports on spontaneous chronic lymphocytic thyroiditis have been published in rhesus macaques. This report describes a case of a Hashimoto-like thyroiditis in a 10-year-old rhesus macaque.

## 2 Animals and methods

### 2.1 Animal provenance

The affected animal was a female, 10-year-old rhesus macaque (*Macaca mulatta*), born at the Paul-Ehrlich-Institut in Langen, Germany, where it lived in an experimental indoor facility. This animal was group-housed in accordance with European and German animal welfare legislation (Directive 2010/63/EU and German “Tierschutzgesetz”). This monkey was used for a preclinical safety study on oncolytic measles virotherapeutics (Völker et al., 2013) 3 months prior to death.

### 2.2 Animal housing

The study animal was housed in a 300 cm × 375 cm × 225 cm steel cage. Large windows allowed the monkey to watch the outside environment. Natural branches, ropes, nets, bedding, mirrors, KONG toys, puzzle feeders, Prima-Hedrons, music, and television were supplied for environmental enrichment. The animal's diet consisted of monkey pellets ad libitum (Trio Munch^®^, Special Diet Services/Mazuri Zoo Foods, Witham, England) in the morning as well as seasonal vegetables and fruits twice weekly in the afternoon. In addition, a mixture of nuts, mealworms, rice, popcorn, and curd was offered to the monkey sporadically.

### 2.3 Clinical history

There were no clinical signs suggestive of a thyroid abnormality.

### 2.4 Necropsy

Necropsy was performed immediately after the death of the animal. Representative organs were fixed in a 4 % formaldehyde solution for 3 d before processing. Paraffin embedding of fixed tissues, preparation of 4 µm sections, and hematoxylin–eosin (H&E) staining were carried out in accordance with standard procedures.

### 2.5 Immunohistochemistry

Immunohistochemistry was performed with 4 µm sections of the tissues fixed in a 4 % formaldehyde solution and embedded in paraffin. After antigen retrieval and blocking, sections were incubated with primary antibodies directed against CD3 and CD20 (Dako-Agilent, Waldbronn, Germany) and were visualized with the avidin–biotin complex technique with alkaline phosphatase using Fast Red (Dako-Agilent, Waldbronn, Germany) as the chromogen.

### 2.6 Laboratory investigations

An electrochemiluminescence immunoassay test system for humans (ECLIA; E170 module, Roche Diagnostics GmbH, Mannheim, Germany) was utilized to evaluate thyroid-stimulating hormone, free triiodothyronine, free thyroxin, and antibodies directed against thyroglobulin, thyroid peroxidase, and thyroid-stimulating hormone receptor in serum (Table 1). The test was performed 2 months after the necropsy with banked serum. The serum of a healthy, age- and gender-matched rhesus macaque was used as a control.

**Table 1:** Electrochemiluminescence immunoassay (ECLIA) thyroid parameters in a rhesus macaque with spontaneous chronic lymphocytic thyroiditis compared to a control animal. mU denotes milli-units, and IU denotes international units.

Parameters tested in serum	Equipment${}^{*}$	Method	Affected monkey	Control monkey
Thyroid-stimulating hormone (TSH)	Modular E170	ECLIA	0.01 mU L${}^{-1}$	0.01 mU L${}^{-1}$
Free triiodothyronine (fT3)	Modular E170	ECLIA	3.4 pg mL${}^{-1}$	4.3 pg mL${}^{-1}$
Free thyroxin (fT4)	Modular E170	ECLIA	0.5 ng dL${}^{-1}$	0.7 ng dL${}^{-1}$
Thyroglobulin antibodies (TG-AB)	cobas 6000 e601	ECLIA	19.6 IU mL${}^{-1}$	18.5 IU mL${}^{-1}$
Thyroid peroxidase antibodies (TPO-AB)	cobas 6000 e601	ECLIA	23.8 IU mL${}^{-1}$	29.7 IU mL${}^{-1}$
Thyroid-stimulating hormone receptor	cobas 6000 e601	ECLIA	0.30 IU L${}^{-1}$	0.79 IU L${}^{-1}$
antibodies (anti-TSHR/TRAb)				

${}^{*}$ Roche Diagnostics GmbH, Mannheim, Germany.

## 3 Results

### 3.1 Necropsy

**Figure 1:**
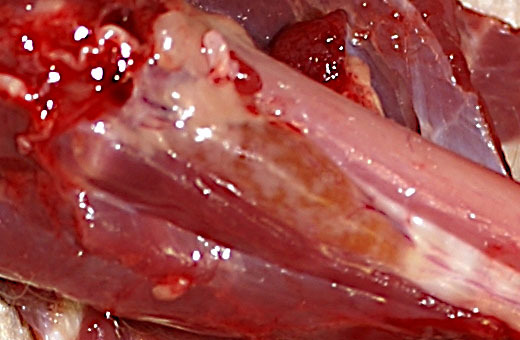
Chronic lymphocytic thyroiditis in a rhesus macaque. The thyroid gland is minimally enlarged, with multifocal to coalescing beige foci.

At necropsy, the thyroid gland was slightly enlarged. Up to 2 mm in diameter, round to oval, confluent, beige spots were present on the surface and cut section (Fig. 1). No pathological abnormalities were detected macroscopically in the remaining organs.

### 3.2 Histopathology

**Figure 2:**
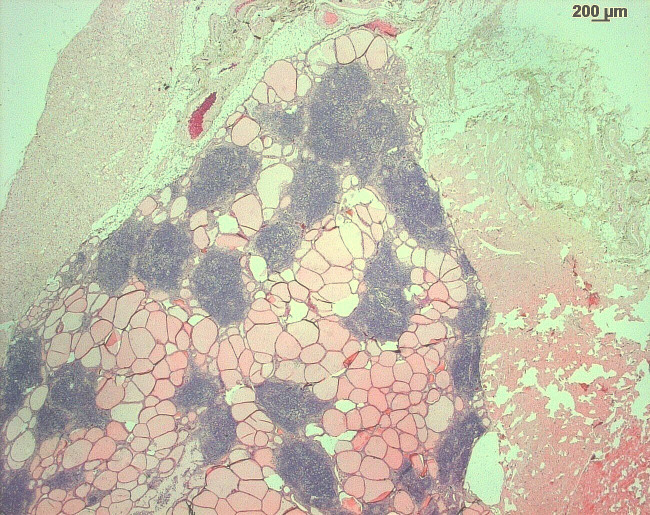
H&E stain of the thyroid gland of a rhesus macaque with chronic lymphocytic thyroiditis: the thyroid gland is disrupted by multifocal to coalescing foci of lymphocytic inflammatory infiltrates.

**Figure 3:**
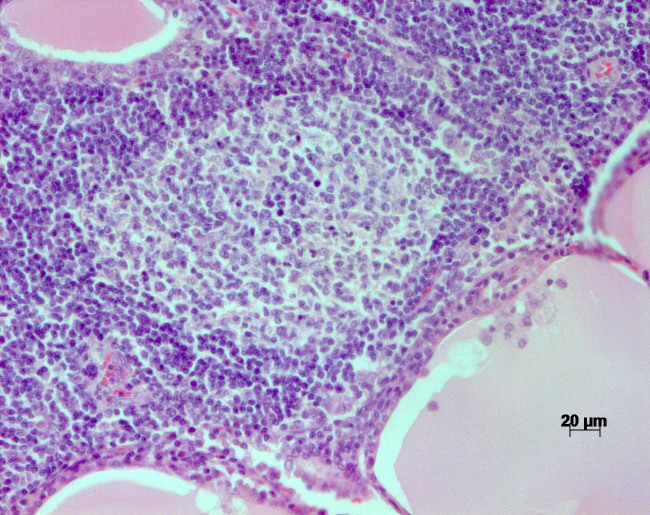
H&E stain of the thyroid of a rhesus macaque with chronic lymphocytic thyroiditis: focal lymphocytic infiltrates with germinal centers within the tissue.

Microscopically, the thyroid gland was disrupted by multifocal areas of lymphocytic infiltration, in part confluent with the interstitium (Fig. 2). Occasionally, areas of lymphocytic infiltration contained lymphoid follicles with distinct germinal centers (Fig. 3). Thyroid follicles ranged from slightly enlarged to small or destroyed, and the latter were frequently associated with regions of lymphocytic inflammation. A small amount of fibrosis was detected within the interstitium of the gland.

**Figure 4:**
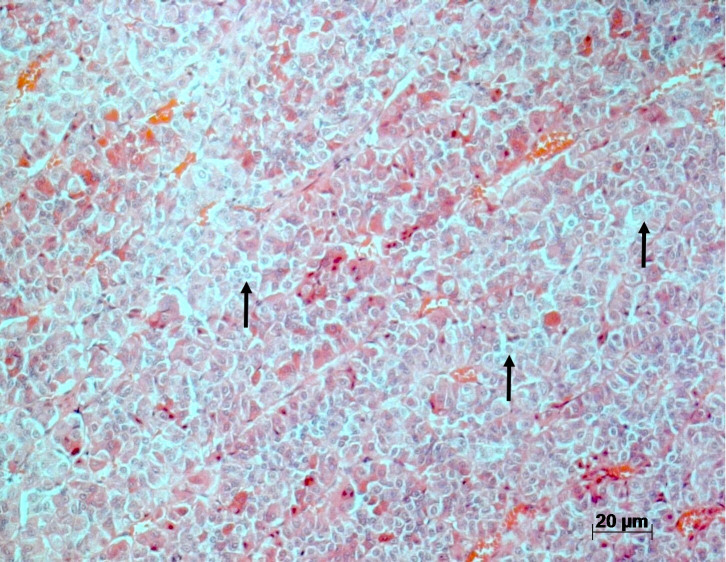
H&E stain of the adenohypophysis of rhesus macaques with chronic lymphocytic thyroiditis. Increased amount of large basophilic cells (arrows).

Within the pituitary adenohypophysis, there were increased numbers of larger cells with pale cytoplasm (basophilic cells) and a hypochromatic cell nucleus, as described by Guzman and Radi in 2007 (Fig. 4).

No histopathological changes were identified in the other organs.

### 3.3 Immunohistochemistry

Using immunohistochemistry, CD-3-positive T cells were demonstrated in the cortex of the follicles, whereas CD-20-positive B cells were found in the center of the follicles.

### 3.4 Laboratory investigations

Using a human ECLIA test system, no significant differences were noted between the affected and the control rhesus macaque (see Table 1).

## 4 Discussion

In humans, Hashimoto's thyroiditis appears to be relatively common, with approximately 5 % of Caucasians impacted. Additionally, females are more frequently affected than males (Pyzik et al., 2015). In contrast, few reports describe spontaneous Hashimoto-like thyroiditis in nonhuman primates. Nevertheless, in the few documented cases (Guzman and Radi, 2007; Levy et al., 1972; AFIP, 2008), females were affected. In humans, clinical conditions may include local manifestations such as compression of cervical structures, goiter, and/or systemic hypothyroidism (Caturegli et al., 2014; Slatosky et al., 2000). Measurement of serum thyroid autoantibodies usually confirms the diagnosis. The three main targets for thyroid antibodies are thyroglobulin, a protein carrier for thyroid hormones; thyroid microsomal antigen (thyroid peroxidase); and the thyroid-stimulating hormone (TSH) receptor (Dayan and Daniels, 1996). In the case presented, there were no clinical signs suggestive of thyroid gland dysfunction. The human ECLIA detection system failed to demonstrate the presence of autoantibodies. In principle, the failure to demonstrate autoantibodies in this case might be due to three different reasons:

1. the test designed for use in humans may not have had adequate cross-reactivity with macaque antibodies;
2. it may be possible that some individual rhesus monkeys, as noted in humans, do not demonstrate increased autoantibodies; and
3. the stage of the disease was too early for the animal to have developed autoantibodies at the time of necropsy.

In humans, a genetic predisposition to thyroid autoimmunity exists and is inherited as a dominant trait (Dayan and Daniels, 1996). In contrast, within the nonhuman primate literature, there is only one assumption of a genetic involvement (Levy et al., 1972). These authors derived their assumption from species- and strain-specific susceptibility to spontaneous thyroiditis.

Histopathological features of chronic lymphocytic thyroiditis in nonhuman primates are similar to those described in humans. However, to our knowledge, autoimmune antibodies in monkeys have only been experimentally induced (Kite et al., 1966); thus, the term “Hashimoto-like” thyroiditis has been applied in this case.

In our case, the rhesus macaque was previously experimentally infected with a recombinant measles virus. It cannot be totally excluded that this vector had an influence on the disease induction and/or progression. However, similar measles virus vectors have previous been used in several human clinical trials (Mühlebach, 2020; Packiriswamy et al., 2020; Msaouel et al., 2018) and no thyroid effects were reported.

Patients with autoimmune (Hashimoto's) thyroiditis have a negative feedback control mechanism within the hypothalamus–pituitary–thyroid axis (Pandiyan and Merrill, 2014). Hypertrophy and hyperplasia of cells producing thyroid-stimulating hormone (TSH) in the adenohypophysis are likely secondary to increased stimulus by the hypothalamic thyrotropin-releasing hormone (TRH) and are considered an adaptive and compensatory response in an attempt to maintain adequate thyroid function (Guzman and Rhadi, 2007), as seen in humans with long-standing primary hypothyroidism and hypertrophy and/or hyperplasia of TSH-producing cells of the pituitary gland (Scheithauer et al., 1985). In a cynomolgus monkey (*Macaca fascicularis*) with Hashimoto's thyroiditis, Guzman and Radi (2007) described epithelial cell hypertrophy and hyperplasia of the adenohypophysis. Hypertrophied cells had an abundant pale basophilic, variable cytoplasmic vacuolation, and were strongly positive for TSH by immunohistochemistry. The normal clinical condition of this monkey and the hypertrophy and hyperplasia of pituitary TSH-producing cells are consistent with normal output of T4 with elevated TSH, as expected in subclinical hypothyroidism. In the case presented, no elevated TSH levels could be confirmed in the laboratory investigation (ECLIA). However, histopathologically, an increased number of larger cells with pale cytoplasm (basophilic cells) and a hypochromatic cell nucleus was observed within the adenohypophysis. Unfortunately, TSH secretion was not performed, as material was not available to perform immunohistochemistry.

In humans, Hashimoto's thyroiditis is sometimes associated with autoimmune-induced alterations in other organs (Cardenas-Roldan et al., 2013). In our case, no pathological findings were identified in the other organs examined – except for the pituitary gland.

## 5 Conclusion

To our knowledge, this study is the first report of spontaneous Hashimoto-like chronic lymphocytic thyroiditis in a rhesus macaque (*Macaca mulatta*). Despite the microscopic similarities to human cases, autoantibodies (thyroglobulin antibodies, thyroid peroxidase antibodies, and thyroid-stimulating hormone receptor antibodies) in this rhesus macaque were not identified using a human ECLIA system.

## Data Availability

Remaining histological slides or paraffin-embedded material is available from the corresponding author upon reasonable request.
